# Dietary Strategies and Novel Pharmaceutical Approaches Targeting Serum ApoA-I Metabolism: A Systematic Overview

**DOI:** 10.1155/2017/5415921

**Published:** 2017-06-12

**Authors:** Lotte Smolders, Jogchum Plat, Ronald P. Mensink

**Affiliations:** Department of Human Biology and Movement Sciences, School of Nutrition and Translational Research in Metabolism (NUTRIM), Maastricht University Medical Center, P.O. Box 616, 6200 MD Maastricht, Netherlands

## Abstract

The incidence of CHD is still increasing, which underscores the need for new preventive and therapeutic approaches to decrease CHD risk. In this respect, increasing apoA-I concentrations may be a promising approach, especially through increasing apoA-I synthesis. This review first provides insight into current knowledge on apoA-I production, clearance, and degradation, followed by a systematic review of dietary and novel pharmacological approaches to target apoA-I metabolism. For this, a systematic search was performed to identify randomized controlled intervention studies that examined effects of whole foods and (non)nutrients on apoA-I metabolism. In addition, novel pharmacological approaches were searched for, which were specifically developed to target apoA-I metabolism. We conclude that both dietary components and pharmacological approaches can be used to increase apoA-I concentrations or functionality. For the dietary components in particular, more knowledge about the underlying mechanisms is necessary, as increasing apoA-I per se does not necessarily translate into a reduced CHD risk.

## 1. Introduction

The global incidence of coronary heart diseases (CHD) is still increasing, which underscores the need for novel and alternative approaches to prevent the initiation and progression of this disease already at an early stage. Since elevated serum low-density lipoprotein cholesterol (LDL-C) concentrations are causally related to CHD, most dietary life style interventions and pharmaceutical treatments to prevent CHD so far are focused on lowering serum LDL-C concentrations. Despite successful intervention possibilities, there is still a substantial residual cardiovascular risk. Therefore, a possibility of further lowering CHD risk is to target multiple metabolic pathways simultaneously [1, 2]. For example, statin treatment, to lower serum LDL-C concentrations, can be combined with other pharmaceutical agents, such as proprotein convertase subtilisin/kexin type 9 inhibitors, which substantially further lower serum LDL-C concentrations [3]. Also, the Niemann-Pick Like Intracellular Cholesterol Transporter 1 inhibitor ezetimibe can be used, which has been shown to further lower the number of myocardial infarctions with 13%, strokes with 14%, and ischemic strokes with 21% [4]. Besides combined interventions to further increase the LDL-C lowering potential, it can be considered to target at the same time other CHD risk parameters including serum high density lipoprotein (HDL) cholesterol (HDL-C), apolipoprotein A-I (apoA-I), triacylglycerol or lipoprotein(a) concentrations, and/or blood pressure [5]. These parameters may be interrelated. An inverse relationship exists, for example, between serum triacylglycerol and HDL-C concentrations. Thus, interventions that change triacylglycerol may therefore also affect HDL metabolism [6]. In this review we will however focus on possibilities to further reduce CHD risk via novel and alternative dietary and pharmacological interventions targeting apoA-I metabolism.

### 1.1. Increasing HDL Functionality by Increasing ApoA-I

So far, interventions specifically targeting to increase serum HDL-C concentrations did not report any protective cardiovascular effect, which has clearly negatively influenced the interest to develop novel interventions to elevate serum HDL-C. However, recent evidence suggests that the focus should be on optimizing HDL functionality instead of elevating circulating serum HDL-C concentrations [7]. By increasing their functionality, HDL particles are able to take up more cholesterol from peripheral tissues, that is, the so-called cholesterol efflux. In addition, a more functional HDL particle will be more antioxidative—in particular by inhibiting LDL oxidation—and more antithrombotic and will have a higher anti-inflammatory and antiapoptotic activity [8]. A wealth of evidence from epidemiological, in vitro, and in vivo studies suggests that higher apoA-I concentrations protect against CHD development [9]. By increasing apoA-I concentrations, the resulting newly produced small HDL particles (i.e., prebeta HDL) will be highly functional, thereby enhancing cholesterol efflux [8]. Indeed, it has been found that apoA-I concentration is the strongest predictor for cholesterol efflux capacity [10]. ApoA-I is the major protein of HDL particles [11] contributing to approximately 33% of the total HDL particle mass and up to 60% of the HDL protein mass [12]. The most likely mechanism explaining the beneficial effects of elevated serum apoA-I concentrations origins from the fact that apoA-I is the ligand for ATP-binding cassette transporter A1 (ABCA1), as such mediating cholesterol efflux from lipid-laden macrophages [8]. Based on this information, Smits et al. wrote a clear plead for strategies to increase serum apoA-I concentrations as the most promising target for enhancing HDL functionality, thereby decreasing cardiovascular disease (CVD) risk [13]. However, lowering CHD risk by increasing endogenous apoA-I production, by decreasing apoA-I degradation, or by providing exogenous apoA-I has for unknown reasons not yet been investigated into great detail. Therefore, the question remains whether specifically targeting apoA-I metabolism is a suitable target to reduce CHD risk.

In this review we will first briefly provide insight into the current knowledge of apoA-I synthesis, clearance, and degradation, followed by a detailed overview of dietary and novel experimental pharmaceutical developments targeting circulating apoA-I concentrations.

## 2. ApoA-I

### 2.1. ApoA-I Synthesis

**Figure 1:**
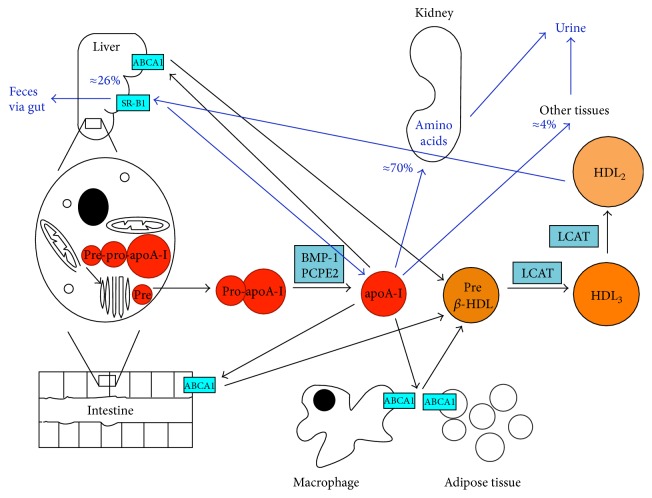
Simplified scheme of the synthesis, metabolism, and clearance of apoA-I. ApoA-I is synthesized in cells of the liver and intestine as pre-pro-apoA-I. After translocation to the endoplasmic reticulum, the preprotein is cleaved of and pro-apoA-I is secreted into blood and lymph. In the circulation, the prosegment is directly cleaved of by Bone Morphogenetic Protein-1 (BMP-1) and Procollagen C-proteinase Enhancer-2 Protein (PCPE2). After this, apoA-I accepts cholesterol and phospholipids from ABCA1, forming a pre-*β* HDL particle. In the circulation, lecithin-cholesterol acyltransferase esterifies (LCAT) the free cholesterol in these pre-*β* HDL particles, forming HDL_3_ and finally HDL_2_, as indicated by the black arrows. After binding of HDL_2_ to SR-B1 on the liver, the cholesterol esters are taken up and lipid-depleted apoA-I is returned to the circulation. These apoA-I-rich particles can again acquire cholesterol and phospholipids or can be cleared from the circulation. Clearance will take place for 70% by the kidney, where apoA-I is broken down into amino acids and ultimately excreted in the urine. 26% of the free apoA-I will be cleared by the liver, and apoA-I catabolic products will then be excreted via biliary secretion into the gut and further digested and absorbed or excreted from the body through the feces. 4% of the free apoA-I will go to other tissues and finally will end up in the urine, as indicated by the blue arrows.

ApoA-I mRNA is expressed in cells of the liver and small intestine [14], where it is translated into a pre-pro-apoA-I protein. The presegment needs cotranslational cleavage [15], which takes place during translocation of the protein into the endoplasmatic reticulum by a signal peptidase [16, 17]. This results in a stable intracellular, pro-apoA-I protein [15], which is secreted into blood and lymph. Directly after secretion of pro-apoA-I, the proprotein is cleaved of by Bone Morphogenetic Protein-1 (BMP-1) and Procollagen C-proteinase Enhancer-2 Protein (PCPE2) (Figure 1) [18, 19]. It is evident that the cleavage of the prosegment is essential for the secretion of newly formed intracellular apoA-I. Deletion of the coding sequence of the prosegment causes accumulation of apoA-I in the cell [20], decreases the efficiency of apoA-I mRNA expression [17], and impairs the secretion of apoA-I into blood and lymph [17, 20]. The cleavage of the proprotein occurs relatively rapid, while the residence time for pro-apoA-I in plasma is only 5.5 hours, in contrast to the residence time for mature apoA-I of 6.5 days [21]. About 4–8% of the circulating apoA-I pool is pro-apoA-I [15, 22, 23]. After cleavage of the prosegment, apoA-I accepts cholesterol and phospholipids from ABCA1 [24] to form a pre-*β* HDL particle (Figure 1). In other words, apoA-I is the starting point for the synthesis of a functional HDL particle and therefore essential for the formation and maturation of novel HDL particles [16]. In the circulation, lecithin-cholesterol acyltransferase esterifies the free cholesterol in these pre-*β* HDL particles, thereby forming HDL_3_ and finally HDL_2_ [25]. The ATP-binding cassette G1 transporter and scavenger receptor class B type 1 (SR-B1) contribute to the cholesterol efflux from peripheral tissues and macrophages to these mature HDL particles. After binding of HDL_2_ to SR-B1 on the liver, cholesterol esters are taken up and lipid-depleted apoA-I is returned to the circulation. These apoA-I-rich lipid-depleted HDL particles can again acquire cholesterol and phospholipids—forming an pre-*β* HDL particle—or can be cleared from the circulation [26].

### 2.2. ApoA-I Clearance

Several organs are involved in apoA-I clearance and degradation [26]. Calculations in rabbits have indicated that renal apoA-I clearance accounts for approximately 68–70% of total apoA-I catabolism. Also in humans, the kidney is the major site for apoA-I clearance [26, 27]. In the kidneys, the uptake of HDL particles is limited, because the intact lipoprotein particles are too large to pass the glomerular filtration barrier. However, newly formed or recycled lipid free apoA-I can pass this barrier. In the proximal tubule of the glomerulus, apoA-I binds the receptors cubilin and megalin [28], which mediate endocytosis and delivery of the protein to the lysosomes [29, 30], resulting in complete degradation of the apoA-I protein. The amino acids can be reused for de novo protein synthesis [31]. While the kidney plays a major role in apoA-I degradation, the liver accounts for 26% of the apoA-I clearance, at least in rats. It is not known how the hepatocytes take up the apoA-I particles, but the apoA-I catabolic products are excreted from the liver via the bile into the gut. In the gut, they are further digested and absorbed or excreted from the body. Other tissues, besides kidney and liver, which are to a lesser extent involved in the degradation of apoA-I, are ovaries, adrenals, and spleen, which secrete apoA-I catabolic products into the urine (Figure 1) [26].

Increasing apoA-I concentrations via reducing apoA-I clearance is for unknown reasons not a subject of investigation. Consequently, it is also not known whether inhibiting apoA-I clearance affects HDL functionality. Therefore, decreasing apoA-I clearance is currently not a target for interventions, whereas elevating de novo apoA-I production certainly is [32].

## 3. Dietary Interventions Affecting ApoA-I Metabolism

It has been clearly shown that dietary components can change serum apoA-I concentrations. We here provide an overview of randomized controlled dietary intervention studies that have examined the effects of whole foods and (non)nutrients on apoA-I concentrations or apoA-I metabolism. Only crossover and parallel studies were included. Potentially relevant studies published before January 2017 were identified by a systematic search of the database PubMed (https://www.ncbi.nlm.nih.gov). The following search terms were used to search in titles and abstracts: (((Clinical Trial[Publication Type]) OR randomized controlled trial[Publication Type])) AND apolipoprotein A^*∗*^[MeSH Terms]. The selection was performed in two steps. First, titles and abstracts were screened. Studies were selected if they met the following inclusion criteria: human intervention study with adults, dietary intervention study, and measurement of apoA-I concentrations. In the second step, full-texts of the selected articles were read to extract fasting or postprandial apoA-I values. Then, a search was performed to find meta-analysis of each food or (non)nutrient group. When a meta-analysis was found, it is included in this review together with the articles identified by us, which were not part of the meta-analysis. Changes in apoA-I concentrations were expressed as percentages, if possible. When percentages were not reported, they were calculated from the mean values as reported in the articles. Furthermore, the list of articles was screened for studies that investigated the effects on cholesterol efflux, apoA-I production rate (PR), or fractional catabolic rate (FCR).

### 3.1. Alcohol

Based on a meta-analysis including 16 studies with in total 374 subjects, Brien et al. concluded that alcohol consumption (women: >15 g alcohol/day; men: >30 g alcohol/day) increased fasting plasma apoA-I concentrations with 10.1 mg/dL (95% CI 7.3–12.9 mg/dL) [33]. A later study, not included in this meta-analysis, also showed a higher fasting apoA-I concentration after alcohol consumption as compared with no alcohol consumption [34]. Moreover, postprandial apoA-I concentrations also increased after alcohol consumption [35]. These effects did not depend on the source (red wine, beer, and Dutch gin) of alcohol [36]. Lavy et al. however reported that red wine increased apoA-I as compared with white wine consumption [37]. Also, Gepner et al. observed that red wine increased apoA-I concentrations as compared with water consumption, but white wine did not significantly change apoA-I concentrations as compared with water or red wine [38]. Furthermore, alcohol consumption not only elevated circulating apoA-I concentrations but also improved HDL functionality as shown by an increased cholesterol efflux capacity [36, 39, 40]. In one study, the kinetics of apoA-I have been examined. It was reported that apoA-I PR increased and apoA-I FCR decreased after alcohol consumption (Table 1) [41].

### 3.2. Boiled and Filtered Coffee, Caffeine, and Tea

In six studies, the effects of boiled or filtered coffee, caffeine, and tea on fasting apoA-I concentrations have been compared. In none of the studies, significant differences in apoA-I concentrations were observed (Table 2) [42–47].

### 3.3. Fatty Acids

In a recent meta-analysis including 104 diets from forty-two well-controlled intervention studies the effects of the various fatty acids on fasting serum apoA-I concentrations were estimated. Effects of fish-fatty acids were not included in that meta-analysis and will be discussed in the next paragraph. A significant increase in serum apoA-I concentrations was found when 1 energy% of carbohydrates was replaced by saturated fatty acids (SFA; 8.4 mg/dL, 95% CI 6.4–10.5), cis-monounsaturated fatty acids (cis-MUFA; 5.5 mg/dL, 95% CI 3.7–7.3), and cis-polyunsaturated fatty acids (cis-PUFA; 2.3 mg/dL, 95% CI 0.1–4.6). cis-MUFA mainly referred to oleic acid and cis-PUFA to linoleic acid plus some *α*-linolenic acid. This meta-analysis further showed that fasting apoA-I concentrations were significantly increased by replacement of 1 energy% from carbohydrates with lauric acid (C12:0; 19.2 mg/dL, 95% CI 14.6–12.7), myristic acid (C14:0; 8.8 mg/dL, 95% CI 0.5–13.1), and palmitic acid (C16:0; 6.5 mg/dL, 95% CI 3.8–9.3), while replacement with stearic acid (C18:0) did not change apoA-I concentrations [48]. For these latter analyses, 88 diets from 34 studies were included. In another meta-analysis based on 17 diets from 10 studies, Brouwer (2016) described the effects of trans-fatty acids (TFA) on circulating fasting apoA-I concentrations. It was reported that replacement of 1 energy% of carbohydrates for total TFA increased apoA-I concentrations (3.3 mg/dL, 95% CI 4.7–1.9). When a difference was made between industrial and ruminant TFA, it was found that replacement with industrial TFA significantly increased fasting apoA-I concentrations (3.3 mg/dL, 95% CI 4.8–1.8), while ruminant TFA did not (4.6 mg/dL, 95% CI: −22.0–12.9). This may be due to a lack of power, since only two studies investigated ruminant TFA. Furthermore, this meta-analysis also showed that replacement of 1 energy% from TFA with SFA increased fasting apoA-I concentrations (2.6 mg/dL, 95% CI 1.4–3.9), while replacement with MUFA did not change apoA-I concentrations and replacement with cis-PUFA decreased fasting apoA-I concentrations (−1.7 mg/dL, 95% CI −2.8–−0.6) (Table 3) [49].

Several studies have examined the effects of the various fatty acids on serum apoA-I metabolism. A TFA diet increased apoA-I FCR as compared with SFA, but the FCR after cis-PUFA consumption did not differ from the TFA or SFA diets. ApoA-I PR was not different between the various diets [52]. Moreover, a cis-PUFA diet did not affect apoA-I FCR [50] and both FCR and PR decreased after low fat consumption compared with high cis-MUFA consumption [51]. In contrast, Labonté et al. have reported that replacing 13 energy% of carbohydrates with cis-MUFA decreased apoA-I FCR with no change in apoA-I PR (Table 3) [53]. The different results between these two studies [51, 53] may have been due to the significant weight loss in the study of Desroches et al., which may have confounded to some extent the effect of MUFA on apoA-I kinetics.

### 3.4. Fish and Fish-Fatty Acids

Most studies investigating the effects of omega-3 fatty acids from fatty fish, mainly eicosapentaenoic acid (EPA) and docosahexaenoic acid (DHA), did not observe any differences in fasting and postprandial apoA-I concentrations [54, 55, 57–66, 68–71, 73–79]. However, in two studies, all in healthy men, fasting apoA-I concentrations decreased after fish oil supplementation. The first study showed lower apoA-I concentrations after pollock oil (rich in EPA) and salmon oil (rich in DHA), but not after tuna oil (rich in DHA) consumption as compared with butter [56]. The second study found lower apoA-I concentrations after EPA oil supplementation compared with DHA oil supplementation [67]. On the other hand, one study found an increase in fasting apoA-I concentrations after a diet high in fish-fatty acids compared with a diet low in fish-fatty acids. In this study, the diets were matched for total fat (Table 4) [72].

Five studies have investigated the effects of fish on fasting apoA-I concentrations. In one study, fatty fish (salmon, rainbow trout, Baltic herring, whitefish, vendace, and tuna) consumption increased apoA-I concentrations compared with lean fish (pike, pike-perch, perch, saithe, and cod) consumption. However, it did not change apoA-I concentrations as compared with lean meat (beef and pork) consumption [83]. The other three studies did not find differences in apoA-I concentrations after fish consumption, of which two compared fatty fish with lean meat [82, 96] and one compared prawns with crab [84]. A limitation of the study of Lindqvist et al. is that participants consumed in total 35 energy% of fat in the herring period and only 10 energy% of fat in the meat period [82], which may have affected apoA-I concentrations. Comparisons between fish and meat consumption are probably not confounded by differences in the intake of the source of protein, as suggested by Gascon et al. In that study, the effects of proteins in lean fish (cod, sole, pollack, and haddock) were compared with those of animal protein (lean beef, pork, veal, eggs, skimmed milk, and milk products). No differences on fasting apoA-I concentrations were found (Table 4) [81].

### 3.5. Fibers

Studies comparing the effects of oat germ, low in fiber, with those of wheat germ, high in fiber, consumption did not find any differences in fasting apoA-I concentrations [80, 85–89]. In four of these studies, it was explicitly reported that the macronutrient composition of the experimental diets was comparable [80, 85, 86, 88]. Mekki et al. observed that a high-fiber diet did not change fasting apoA-I concentrations as compared with a low fiber diet [90]. On the other hand, decreased fasting apoA-I concentrations were found after a high *β*-glucan and psyllium diet as compared with a low fat, low-cholesterol control diet [91]. The water-soluble fiber arabinoxylan also decreased fasting apoA-I concentrations as compared with the control diet, which had a similar macronutrient composition [92]. Furthermore, no differences in fasting apoA-I concentrations were observed between the soluble and insoluble forms of* P*.* ovate* [93]. The water-soluble fiber *β*-glucan did not affect fasting apoA-I concentrations [94]. Furthermore, wheat germ consumption increased fasting apoA-I concentrations compared with flaxseed consumption (Table 5) [95].

### 3.6. Nuts

In one short-term study, walnut consumption significantly increased fasting serum apoA-I concentrations [97], but these effects were not found in two longer-term studies [98, 99]. Almond consumption did also not affect fasting apoA-I concentrations [100, 101]. Likewise, hazelnuts [102, 103] and pistachio nuts did not change fasting apoA-I concentrations [104]. A limitation of some of the studies is that not all experimental diets were matched for differences in fat and fatty acid composition. In some of these studies, the diets containing nuts provided more energy from fat than the control diets [99–102]. Furthermore, the nut diets were sometimes also lower in SFA and higher in PUFA than the control diets [99, 101]. Although these differences in nutrient intakes are inherent to consuming more nuts, it is not likely that the effects observed are due to minor component in nuts, since fatty acids increase apoA-I concentrations as compared with carbohydrates [48]. However, most other studies that used a control diet with similar fat and fatty acid composition did also not find any effects of the consumption of nuts on apoA-I concentrations (Table 6) [98, 103, 104].

### 3.7. Plant Sterols and Stanols

Most studies examining the effects of plant sterols on serum lipids did not demonstrate an effect of plant sterols on fasting apoA-I concentrations [106, 107, 109–114]. In one study, comparing olive oil and olive oil with plant sterol esters and sunflower oil with plant sterol esters, fasting apoA-I concentrations increased when plant sterol esters were consumed together with olive oil, but apoA-I concentrations were comparable during the other two interventions [108]. Furthermore, one study showed an increase in fasting apoA-I concentrations comparing 3 months of prudent diet consumption (National Cholesterol Education Program) with added plant sterols, with prudent diet consumption alone [111]. One study examined the effects of plant stanols on fasting apoA-I concentrations and found increased apoA-I concentrations comparing 6 weeks of sitostanol consumption with no sitostanol consumption [105]. Finally, no changes in apoA-I PR and FCR were found after plant sterol or stanol consumption (Table 7) [105, 110].

### 3.8. Soy Proteins or Isoflavones Isolated from Soy

Studies investigating the effects of soy protein on fasting apoA-I concentrations showed inconsistent outcomes. Eight studies using different amounts of soy protein for 3 weeks till 3 months did not find changes in fasting apoA-I concentrations [115, 116, 118–121, 123–125]. On the other hand, in one study products containing soy protein increased fasting apoA-I concentrations as compared with products containing casein [117], while in another study products with soy protein decreased fasting apoA-I as compared with products containing casein [122]. Furthermore, two studies found different effects of various soy products on fasting apoA-I concentrations [126, 127]. Soy-milk increased apoA-I concentrations as compared with soy nuts and soy flour, but no differences were found as compared with animal protein [126]. Soy nut and soy protein consumption increased apoA-I concentrations as compared with the control group without soy [127]. Two studies have investigated the effects of isoflavones isolated from soy on apoA-I concentrations and showed no effect on fasting [128, 129] and postprandial apoA-I concentrations (Table 8) [129].

### 3.9. Others

Many other products and food components have been studied for their effects on apoA-I concentrations. In most of these studies, which included eggs [130], dried garlic [131, 134], beta-carotene [132], phytochemicals with cytochrome P-450-inducing activity [133], magnesium [135], eggplant [136], dry beans [137], kiwifruits [138], and polyphenols [139], no effects on fasting apoA-I concentrations were observed. In addition, sphingolipids did not change postprandial apoA-I concentrations [140]. On the other hand, red grape juice [141], a mixture of citrus flavonoids and tocotrienols [142], vitamin D supplementation [143, 144], vitamin D plus calcium supplementation [144], theobromine [145], orange juice [146], and a high dose of grape pomace and omija fruit [147] all increased fasting apoA-I concentrations (Table 9).

## 4. Pharmacological Approaches Targeting ApoA-I Metabolism

Although not always specifically developed for this purpose, several well-known drugs like statins [7, 167] and CETP inhibitors [168–171] affect serum apoA-I concentrations. However, since this review focuses on novel strategies to increase serum apoA-I concentrations, we here describe only approaches that are currently in development and are specifically designed to target a change in apoA-I metabolism. Potentially relevant studies published before January 2017 were identified by a systematic search of the database PubMed (https://www.ncbi.nlm.nih.gov). The following search terms were used to search in titles and abstracts: (Pharmacological AND approaches AND apoA-I). First, all abstracts were screened and the pharmacological approaches were divided into three categories: apoA-I mimetics, apoA-I infusions, and others. Second, a new search was performed with the search terms: (apoA-I mimetics AND apoA-I infusions AND RVX-208 AND LCAT infusion AND clinical trial) to select all studies published before January 2017 that investigate apoA-I mimetics, apoA-I infusions, and RVX-208 in humans.

### 4.1. ApoA-I Mimetics

ApoA-I mimetics are small amphipathic peptides that resemble apoA-I in biological function and structure [172]. These mimetics are not the intact apoA-I protein, but small fragments of the protein with certain biological functions. These small peptides can be given orally or can be infused [15, 173]. To prevent digestion in the gastrointestinal tract, mimetics are made from D-amino acids, which are resistant to human gastrointestinal proteolytic enzymes [14]. Over the years, several mimetics have been produced, but none of them has all the antiatherogenic functions of apoA-I. However, combining several mimetics can be a theoretical approach to mimic all antiatherosclerotic properties of apoA-I [174]. The only mimetic that has been tested in humans is D-4F. When 50 patients with coronary artery disease received a single oral dose (30, 100, 300, and 500 mg) of this mimetic, the two highest doses increased the anti-inflammatory activity of the HDL fraction. However, no changes in lipids or lipoprotein concentrations were seen. D-4F was shown to be safe and well tolerated (Table 10) [148]. Unfortunately, the effects of D-4F on cholesterol efflux in humans have not yet been investigated.

### 4.2. ApoA-I Infusions

Besides apoA-I mimetics, apoA-I itself, either by using delipidated HDL or by using delipidated HDL combined with phospholipids, can be infused directly into the circulation. The theoretical advantage of using apoA-I or apoA-I-phospholipid complexes instead of using apoA-I mimetics is that the apoA-I protein is completely intact and still possesses all its biological functions and might therefore have a larger atheroprotective effect. So far, three different forms of apoA-I have been tested, that is, apoA-I Milano (MDCO-216), CSL-111/CSL112, and CER-001.

#### 4.2.1. ApoA-I Milano

In a randomized human controlled trial, 47 patients with acute coronary syndromes received for 5 weeks one infusion of placebo or recombinant apoA-I Milano/phospholipid complex (ETC-216) at 15 or 45 mg/kg per week. At the end of the study a significant reduction in atheroma volume was found in the high dose group [149]. This reduction in atheroma volume was accompanied by a reduction in external elastic membrane volume of the artery, but not with a change in lumen volume [175]. Recently, in a randomized controlled study, patients with stable coronary artery disease received 5 doses of 10, 20, 30, and 40 mg/kg MDCO-216 infusion. This resulted in a dose-dependent increase in apoA-I concentrations and a dose-dependent shift from small- to large-sized HDL particles [150]. Moreover, a profound increase in ABCA1-mediated cholesterol efflux was observed [151]. However, very recently the MILANO-PILOT study failed to slow down the regression of coronary atherosclerosis with 5 weekly infusions of 20 mg/kg MDCO-216 in 120 patients with acute coronary syndromes. In fact, significant reductions in HDL-C and apoA-I concentrations were observed, while there were no effects found on percent atheroma volume and total atheroma volume (Table 10) [152].

#### 4.2.2. CSL-111/CSL112

In one clinical study, 40 and 80 mg/kg CSL-111 were infused once a week for one month in 183 patients elected for coronary angiography. Treatment of the high dose group (80 mg/kg) was discontinued early, because some of the patients exceeded the upper level of alanine aminotransferase by 100-fold. The low dose group (40 mg/kg) showed a significant reduction in atheroma volume. However, this reduction was not significantly different from the decrease in the placebo group [176]. After this, the further development program of CSL-111 was discontinued because of the unfavorable hepatic abnormalities. As a follow-up, one phase I study has been performed using CSL112, which is a similar compound, but postulated without effects on liver function. In this study, a single dose (5, 15, 40, 70, 105, or 135 mg/kg) or multiple doses for 4 weeks (3.4 or 6.7 g once a week or 3.4 g twice a week) of CSL112 was administrated intravenously to healthy volunteers. Both the single and multiple doses of CSL112 dose dependently increased serum apoA-I and serum HDL-C concentrations. Moreover, also pre-*β* HDL particle concentrations and cholesterol efflux capacity were increased. In the single dose study, dose-dependent effects were found on HDL-C [153, 154]. Recently, two studies showed that CSL112 was indeed safe for human consumption, with no effects on liver function parameters [155, 156]. In the first study, patients with atherosclerosis were given infusions of 1.7, 3.4, and 6.8 g CSL112 or placebo. The CSL112 infusions resulted in a dose-dependent increase in apoA-I and total cholesterol efflux [155]. In the second study patients with myocardial infarction received infusions of 2 or 6 g CSL112 or placebo for 4 weeks. Here also a dose-dependent increased in HDL-C, apoA-I, and cholesterol efflux was shown (Table 10) [156].

#### 4.2.3. CER-001

In one clinical study, 417 patients with acute coronary syndromes were randomized for 6 weekly infusions of 3, 6, and 12 mg/kg CER-001 or placebo. No changes in atheroma volumes were found. It was speculated that a higher dose or a different patient group would have shown more positive results [157]. In a recent human study, 9 infusions of 8 mg/kg CER-001 were given twice weekly for 28 days to 7 patients with familial hypoalphalipoproteinemia, who were severely deficient in HDL. In this patient group, CER-001 significantly increased serum apoA-I and HDL-C concentrations and reduced atherosclerotic lesion size, measured using Magnetic Resonance Imaging. Moreover, an increase in cholesterol efflux from macrophages and a higher fecal neutral sterol excretion was seen, which may indicate improved RCT [158]. Additionally, 12 biweekly infusions with 8 mg/kg CER-001 showed increased apoA-I concentrations, a decrease in vessel wall area, and a trend toward a reduction in vessel wall thickness [159]. Recently, a study evaluated the effects of 3 mg/kg CER-001, in patients with atherosclerotic carotid artery disease, and showed increased apoA-I concentrations, with a simultaneously increased cholesterol efflux capacity [160]. Unfortunately, preliminary data of a recent clinical trial in patients with coronary atherosclerosis did not show beneficial effects of CER-001 on atheroma volume and LDL-C [161] (Table 10).

### 4.3. Others

#### 4.3.1. RVX-208

The first class of compounds affecting apoA-I metabolism refers to the apoA-I transcriptional upregulator RVX-208. RVX-208 is an oral, small synthetic quinazoline molecule, which binds bromo- and extra terminal (BET) proteins and upregulates apoA-I gene transcription via an epigenetic mechanism.

In the first human clinical trial, 18 healthy subjects received varying and multiple doses (1 to 20 mg/kg per day) of RVX-208 or placebo for 7 days. Plasma apoA-I concentrations were increased, and more importantly, an increase in pre-*β*1-HDL concentrations and a higher ABCA1-mediated cholesterol efflux was demonstrated [162]. The outcome of the recent phase 2 randomized placebo-controlled clinical ASSERT trial, evaluating the effect of RVX-208 on serum apoA-I concentrations and CHD risk in human, was less positive. In that study, 299 patients with stable coronary artery disease received placebo or RVX-208 at three different dosages (50, 100, and 150 mg) twice daily for 12 weeks. Only a nonsignificant increase in serum apoA-I concentrations was found. Unfortunately, HDL functionality and cholesterol efflux capacity were not studied [163]. A second study using RVX-208 is the phase 2b clinical trial SUSTAIN. In this trial, 172 statin-treated patients (Rosuvastatin or Atorvastatin) with low serum HDL-C concentrations were treated with 200 mg/day RVX-208 for 24 weeks. Both serum apoA-I concentrations and HDL particle numbers increased significantly. Furthermore, RVX-208 was found to be safe for oral use [164]. In another phase 2 clinical trial, the ASSURE study, 323 statin (Rosuvastatin or Atorvastatin) treated patients with coronary artery disease and low serum HDL-C concentrations received 100 mg RVX-208 twice daily for 26 weeks. However, no significant reductions in atheroma volume or increases in HDL-C and apoA-I concentrations were seen [32]. Finally, a recent study in subjects with prediabetes showed that 100 mg RVX-208 for 29–33 days did not increase HDL-C and apoA-I concentrations, while it increased the concentration of medium-sized HDL and decreased the concentration of small-sized HDL particles. Furthermore, RVX-208 delayed and reduced oral glucose absorption and endogenous glucose production (Table 10) [165].

#### 4.3.2. LCAT Infusion

The first human study investigating the effects of lecithin-cholesterol acyltransferase (LCAT) infusion investigated only one patient with familial LCAT deficiency. Recombinant human LCAT was infused 3 times for 1 hour in a dose optimization phase (0.3, 3.0, and 9.0 mg/kg) and after this 1 to 2 weekly infusions were given of 3.0 or 9.0 mg/kg for 7 months. LCAT infusion improved renal function, increased apoA-I, HDL-C, and to a lesser extent LDL-C. Furthermore, after infusion, postprandial triacylglycerol concentrations decreased [166]. These results are promising; however, before drawing conclusions about LCAT infusion clinical trials including more patients should be done.

## 5. Conclusion

Alcohol consumption increases fasting apoA-I concentrations and may improve cholesterol efflux, possibly via increasing apoA-I PR and decreasing FCR. Further, replacement of carbohydrates for SFA, cis-MUFA, cis-PUFA, and TFA increases fasting apoA-I concentrations. The effects of the various SFA are different, since lauric, palmitic, and myristic acids increase apoA-I concentrations, while stearic acid does not. The different fatty acids affect apoA-I metabolism differently, but results are conflicting. Therefore more studies are needed to better understand the effects of the various macronutrients on apoA-I kinetics.

Coffee, caffeine, tea, omega 3 fatty acid, fish, nuts, plant sterol and stanol, different soy proteins, and isoflavones isolated from soy do not change fasting apoA-I concentrations. Moreover, the effects of the various types of fibers may be different; the consumption of diets rich in wheat germ did not modify apoA-I concentrations, while the consumption of diets rich in psyllium, arabinoxylan, and flaxseed may decrease fasting apoA-I concentrations. However, these types of fibers have only been examined in a limited number of studies. Therefore, we conclude that fiber consumption does not have a profound impact on fasting apoA-I concentrations.

Finally, five other food components showed a promising increase in fasting apoA-I concentrations: citrus, vitamin D, theobromine, orange juice, and a high dose of grape pomace and omija fruit. However, these findings need to be confirmed in future studies. Additional research is also needed to examine the effects of these products or food components not only on apoA-I kinetics, but also on HDL functionality.

Overall, all three categories of pharmacological approaches showed that targeting apoA-I concentrations and/or HDL functionality by a pharmacologic approach can increase apoA-I functionality and might improve CHD risk markers, including vessel wall characteristics and inflammation. The mimetic D-4F is promising, but clinical studies are required to investigate the effects on HDL functionality. The CSL112 and LCAT infusions are the most promising of the infusion therapies, but studies are needed to investigate the effects of CSL112 on CHD risk markers, including vessel wall characteristics and inflammation, and LCAT infusions need to be investigated in clinical trials with more patients. Unfortunately, recent clinical studies showed no improvement in CHD risk markers after apoA-I Milano, RVX-208, or CER-001 therapy.

Although we cannot exclude that we have missed studies during the systematic searches and studies with positive results are overrepresented, we conclude that both dietary components and pharmacological approaches can be used to increase apoA-I concentrations. For the dietary components in particular, more knowledge about underlying mechanisms is necessary, as increasing apoA-I per se does not necessarily translate into a reduced CHD risk.

## Figures and Tables

**Table 1:** Effect of alcohol consumption on apoA-I concentrations, HDL functionality, and apoA-I kinetics.

First author, year	Food component/product	Study design and duration	Participants	Intake	Effects
Brien et al. (2011) [33]	Alcohol	Meta-analysis of 16 studies till 2009 (i) RCT with 2 arms (ii) before versus after >1 week	374 subjects	Women > 15 g/day Men > 30 g/day	(i) 10.1 mg/dL (95% CI 7.3–12.9) ↑ in fasting plasma apoA-I concentrations

Lavy et al. (1994) [37]	Red versus white wine	RCT parallel 2 weeks	20 healthy men	44 g alcohol/day	(i) 12.0% ↑ in fasting plasma apoA-I concentrations comparing red wine with white wine

van der Gaag et al. (1999) [35]	Red wine versus beer versus Dutch gin versus water	RCT crossover 3 weeks	11 healthy men	40 g alcohol/day from red wine, beer, or Dutch gin	(i) 8.2% ↑ in fasting serum apoA-I concentrations comparing alcohol with water^*∗*^ (ii) 9.2% ↑ in postprandial serum apoA-I concentrations comparing alcohol with water^*∗*^ (iii) No differences between the different beverages

van der Gaag et al. (2001) [36]	Red wine versus beer versus Dutch gin versus water	RCT crossover 3 weeks	11 healthy men	40 g alcohol/day from red wine, beer, or Dutch gin	(i) 10% ↑ in fasting plasma apoA-I concentrations comparing alcohol with water^*∗*^ (ii) 6.2% ↑ in cholesterol efflux comparing alcohol with water^*∗*^ (iii) No differences between the different beverages

Beulens et al. (2004) [39]	Whisky versus water	RCT crossover 17 days	23 healthy men	40 g alcohol/day	(i) 6.2% ↑ in fasting plasma apoA-I concentrations (ii) 17.5% ↑ in cholesterol efflux

Kralova Lesna et al. (2009) [40]	Beer versus nonalcoholic beverage	RCT crossover 4 weeks	13 healthy men	36 g alcohol/day	(i) 7.5% ↑ in fasting plasma apoA-I concentrations^*∗*^ (ii) 8.0% ↑ in fasting cholesterol efflux^*∗*^

Gepner et al. (2015) [38]	Red versus white wine versus water	RCT parallel 2 years	195 patients with diabetes mellitus type 2	17 g alcohol/day	(i) 2.3% ↑ in fasting plasma apoA-I concentrations comparing red wine with water^*∗*^ (ii) No difference between white wine and water or red wine

Chiva-Blanch et al. (2013) [34]	Red wine versus dealcoholized red wine versus gin	RCT crossover 4 weeks	67 men at high CVD risk	30 g alcohol/day	(i) 12.5% and 12.6% ↑ in fasting plasma apoA-I concentrations comparing dealcoholized red wine with red wine and gin, respectively^*∗*^

Gottrand et al. (1999) [41]	Red wine versus nonalcoholic beverage	RCT 4 weeks	5 healthy men	50 g alcohol/day	(i) 20% ↑ in plasma apoA-I pool (ii) 10% ↑ in PR (iii) 6% ↓ in FCR

^*∗*^Percentages calculated from the mean values; PR: production rate; FCR: fractional catabolic rate.

**Table 2:** Effect of boiled and filtered coffee, caffeine, and tea on fasting apoA-I concentrations.

First author, year	Food component/product	Study design and duration	Participants	Intake	Effects
Aro et al. (1987) [43]	Boiled versus filtered coffee versus tea	RCT crossover 4 weeks	42 hypercholesterolemic subjects	8 cups/day	(i) No differences in serum apoA-I concentrations

Aro et al. (1990) [42]	Boiled versus filtered coffee	RCT crossover 4 weeks	41 healthy subjects	2–14 cups/day	(i) No differences in serum apoA-I concentrations

van Dusseldorp et al. (1991) [47]	Filtered versus unfiltered coffee versus no coffee	RCT parallel 79 days	64 healthy subjects	6 cups/day	(i) No differences in serum apoA-I concentrations

Burr et al. (1989) [44]	Decaffeinated versus no coffee	RCT crossover 4 weeks	54 healthy subjects	>5 cups/day	(i) No differences in plasma apoA-I concentrations

Davies et al. (2003) [45]	Black tea versus caffeine versus caffeine free placebo	RCT crossover 3 weeks	15 mildly hypercholesterolemic subjects	5 cups/day	(i) No differences in plasma apoA-I concentrations

Mozaffari-Khosravi et al. (2009) [46]	Sour tea versus black tea	RCT parallel 1 month	53 patients with diabetes mellitus type 2	2 cups/day	(i) No differences in serum apoA-I concentrations

**Table 3:** Meta-analysis showing the effects of fatty acids on fasting apoA-I concentrations and studies showing effects of fatty acids on apoA-I kinetics.

First author, year	Food component/product	Study design and duration	Participants	Intake	Effect
Mensink (2016) [48]	Replacement of carbohydrates (carbs) for SFA, MUFA, or PUFA	Meta-analysis of 42 studies till Dec 2013 Daily controlled RCT parallel and crossover >13 days	Healthy subjects	1% of dietary energy	(i) 8.4 mg/dL (95% CI 6.4–10.5) ↑ in fasting apoA-I concentrations replacing carbs with SFA (ii) 5.5 mg/dL (95% CI 3.7–7.3) ↑ in fasting apoA-I concentrations replacing carbs with MUFA (iii) 2.3 mg/dL (95% CI 0.1–4.6) ↑ in fasting apoA-I concentrations replacing carbs with PUFA

Mensink (2016) [48]	Replacement of carbs for lauric acid (C12:0), myristic acid (C14:0), palmitic acid (C16:0), or stearic acid (C18:0)	Meta-analysis of 34 studies till Dec 2013 Daily controlled RCT parallel and crossover >13 days	Healthy subjects	1% of dietary energy	(i) 19.2 mg/dL (95% CI 14.6–12.7) ↑ in fasting apoA-I concentrations replacing carbs with lauric acid (ii) 8.8 mg/dL (95% CI 0.5–13.1) ↑ in fasting apoA-I concentrations replacing carbs with myristic acid (iii) 6.5 mg/dL (95% CI 3.8–9.3) ↑ in fasting apoA-I concentrations replacing carbs with palmitic acid (iv) No difference in apoA-I concentrations replacing carbs with stearic acid

Brouwer (2016) [49]	Replacement of trans-fatty acids (TFA) for carbs	Meta-analysis of 10 studies till Sep 2014 Daily controlled RCT parallel and crossover >13 days	Healthy subjects	1% of dietary energy	(i) 3.3 mg/dL (95% CI 4.7–1.9) ↑ in fasting apoA-I concentrations replacing carbs with TFA (ii) 2.6 mg/dL (95% CI 1.4–3.9) ↑ in fasting apoA-I concentrations replacing TFA with SFA (iii) No difference in apoA-I concentrations replacing TFA with MUFA (iv) 1.7 mg/dL (95% CI −2.8–−0.6) ↓ in fasting apoA-I concentrations replacing TFA with PUFA

Ginsberg et al. (1994) [50]	Average American diet versus PUFA enriched diet	RCT parallel 6 weeks	21 healthy men	MUFA: 14 versus 8 energy% PUFA: 7 versus 13 energy%	(i) No difference in apoA-I FCR

Desroches et al. (2004) [51]	Low fat diet versus high MUFA diet	RCT parallel 6-7 weeks	18 healthy men	Fat: 25.8 versus 40.1 energy% MUFA: 13.3 versus 22.5 energy%	(i) 31% ↓ PR after low fat compared with high MUFA diet (ii) 22% ↓ FCR after low fat compared with high MUFA diet

Matthan et al. (2004) [52]	Soybean oil (PUFA) versus margarine (TSA) versus butter (SFA)	RCT crossover 5 weeks	8 hypercholesterolemic women	2/3 of the total fat intake	(i) 11% ↑ FCR after margarine compared with butter (ii) No difference in PR between the diets

Labonté et al. (2013) [53]	Carbohydrates versus MUFA	RCT parallel 4 weeks	16 dyslipidemic subjects	13 energy%	(i) 5.6% ↑ in FCR after carbohydrate compared with MUFA consumption (ii) No difference in PR between the diets

SFA: saturated fatty acids, MUFA: monounsaturated fatty acids, PUFA: polyunsaturated fatty acids, TFA: trans-fatty acids, PR: production rate, and FCR: fractional catabolic rate.

**Table 4:** Effect of fish oil or fish on apoA-I concentrations.

First author, year	Food component/product	Study design and duration	Participants	Intake	Effect
Schectman et al. (1988) [54]	Low versus high fish oil versus safflower oil capsules	RCT crossover 1 month	13 patients with noninsulin-dependent diabetes mellitus type 2	4.0 versus 7.5 g omega-3/day 12 g safflower oil/day	(i) No differences in fasting plasma apoA-I concentrations

Wilt et al. (1989) [55]	Fish oil versus safflower oil capsule	RCT parallel 12 weeks	38 healthy men	20 g/day	(i) No differences in fasting plasma apoA-I concentrations

Childs et al. (1990) [56]	Pollock oil (EPA) versus tuna (DHA) versus salmon (DHA) versus butter capsule	RCT crossover 3 weeks	8 healthy men	EPA: 11.7, 5.4, and 6.1 g/day DHA: 3.1, 15.5, and 7.7 g/day	(i) 22.0% ↓ in fasting plasma apoA-I concentrations comparing pollock oil with control (ii) 14.0% ↓ in fasting plasma apoA-I concentrations comparing salmon oil with control (iii) No differences in fasting plasma apoA-I concentrations comparing tuna oil with control

DeLany et al. (1990) [57]	Low versus high fish oil versus margarine (similar macronutrient composition)	RCT parallel 5 weeks	15 healthy men	0, 5, and 20 g fish oil/day with 0, 2, and 8 g omega-3/day	(i) No differences in fasting serum apoA-I concentrations

Levinson et al. (1990) [58]	Fish oil versus vegetable oil capsule	RCT parallel 6 weeks	16 mild hypertensive patients	50 g oil/day, 9 g EPA, and 6 g DHA	(i) No differences in fasting serum apoA-I concentrations

Mori et al. (1990) [59]	Fish oil versus no fish oil	RCT parallel 3 weeks	22 insulin-dependent diabetic men	2.7 g EPA and 1.7 g DHA/day	(i) No differences in fasting serum apoA-I concentrations

Boønaa et al. (1992) [60]	EPA and DHA oil versus corn oil capsule	RCT parallel 10 weeks	156 healthy subjects	5.1 g/day	(i) No differences in fasting serum apoA-I concentrations

Richter et al. (1992) [61]	Omega-3 versus omega-6 capsule	RCT crossover 3 weeks	26 healthy men	5.0 g/day	(i) No differences in fasting plasma apoA-I concentrations

Tatò et al. (1993) [62]	EPA and DHA versus olive oil capsules	RCT crossover 4 weeks	9 patients with familial hyperlipidemia	3.0 and 4.5 g EPA and DHA/day	(i) No differences in fasting serum apoA-I concentrations

Zampelas et al. (1994) [63]	SFA oil versus corn oil versus fish oil capsule	RCT crossover 1 day	12 healthy men	40 g/day	(i) No differences in postprandial serum apoA-I concentrations

Eritsland et al. (1995) [64]	Fish oil capsule versus no capsule	RCT parallel 9 months	511 patients with coronary artery disease	4 g/day: 3.4 g EPA and DHA	(i) No differences in fasting serum apoA-I concentrations

Herrmann et al. (1995) [65]	Omega-3 versus rapeseed oil capsule	RCT parallel 4 weeks	53 patients with coronary artery disease	8.5 g/day	(i) No differences in fasting plasma apoA-I concentrations

Hamazaki et al. (1996) [66]	DHA versus control oil capsule	RCT parallel 13 weeks	24 healthy subjects	1.5–1.8 g/day	(i) No differences in fasting serum apoA-I concentrations

Grimsgaard et al. (1997) [67]	EPA versus DHA versus corn oil capsule	RCT parallel 7 weeks	234 healthy men	EPA: 3.8 g/day DHA: 3.6 g/day Corn oil: 4.0 g/day	(i) 5.0% ↓ in fasting serum apoA-I concentrations comparing EPA with corn oil^*∗*^ (ii) No differences in fasting serum apoA-I concentrations comparing DHA with corn oil^*∗*^

Sorensen et al. (1998) [68]	Fish oil versus sunflower oil margarine	RCT parallel 4 weeks	47 healthy subjects	4.0 g/day	(i) No differences in fasting plasma apoA-I concentrations

Buckley et al. (2004) [69]	EPA versus DHA versus olive oil capsules	RCT parallel 4 weeks	42 healthy subjects	EPA: 4.8 g/day DHA: 4.9 g/day	(i) No differences in fasting plasma apoA-I concentrations

Calabresi et al. (2004) [70]	Omega-3 versus placebo capsule	RCT crossover 8 weeks	14 patients with familial hyperlipidemia	EPA: 1.88 g/day DHA: 1.48 g/day	(i) No differences in fasting plasma apoA-I concentrations

Shidfar et al. (2003) [71]	Omega-3 versus placebo	RCT parallel 10 weeks	68 hyperlipidemic patients	1 g/day	(i) No differences in fasting serum apoA-I concentrations

Li et al. (2004) [72]	High versus low fish-fatty acids diet	RCT parallel 24 weeks	22 healthy subjects	30 energy% of fat	(i) 14.0% ↑ in fasting plasma apoA-I concentrations comparing high with low fish-fatty acids consumption

Goyens and Mensink (2006) [73]	ALA versus EPA and DHA capsule	RCT parallel 6 weeks	37 elderly healthy subjects	ALA 6.8 g/day, EPA, and DHA: 1.05 + 0.55 g/day	(i) No differences in fasting serum apoA-I concentrations

De Roos et al. (2008) [74]	Fish oil versus high oleic sunflower oil capsule	RCT parallel 6 weeks	81 healthy subjects	3.5 g/day: 700 mg EPA and 560 mg DHA	(i) No differences in fasting serum apoA-I concentrations

Shidfar et al. (2008) [75]	Omega-3 versus 300 mg SFA, 100 mg MUFA, and 600 mg linoleic acid capsule	RCT parallel 10 weeks	50 patients with diabetes mellitus type 2	2 g/day: 520 mg EPA and 480 mg DHA	(i) No differences in fasting serum apoA-I concentrations

Maki et al. (2011) [76]	Omega-3 versus soy oil	RCT crossover 6 weeks	31 patients with primary, isolated hypercholesterolemia	4 g/day	(i) No differences in fasting serum apoA-I concentrations

Ooi et al. (2012) [77]	Therapeutic lifestyle change diet low versus high in fish (capsule)	RCT parallel 24 weeks	20 healthy subjects	EPA and DHA: 1.23 g/day versus 0.27 g/day	(i) No differences in fasting plasma apoA-I concentrations

Song et al. (2013) [78]	High omega-3 and low omega-6 versus low omega-3 and high omega-6 fatty acid diet	RCT crossover 1 day	8 healthy and 8 hypertriacylglycerolemic subjects	0.97 versus 8.80 n-6/n-3 ratio	(i) No differences in postprandial serum apoA-I concentrations

Oliveira et al. (2014) [79]	Fish oil versus soya oil supplements	RCT parallel 24 weeks	83 HIV-infected subjects on antiretroviral therapy	3 g/day: 540 mg EPA and 360 mg DHA	(i) No differences in postprandial serum apoA-I concentrations

Stewart et al. (1990) [80]	Mackerel versus lean meat	RCT crossover 6 weeks	84 healthy male subjects	135 g/day	(i) No differences in fasting serum apoA-I concentrations

Gascon et al. (1996) [81]	Lean fish (cod, sole, pollack, and haddock) versus animal protein (lean beef, pork, veal, eggs, skimmed milk, and milk products)	RCT crossover 4 weeks	14 premenopausal healthy women	69–71 energy% protein	(i) No differences in lean fish on fasting plasma apoA-I concentrations

Lindqvist et al. (2007) [82]	Herring versus lean meat (pork and chicken)	RCT crossover 4 weeks	13 healthy obese subjects	50 g/day 5 days/week	(i) No differences in fasting plasma apoA-I concentrations

Erkkilä et al. (2008) [83]	Fatty (salmon, rainbow trout, Baltic herring, whitefish, vendace, and tuna) versus lean fish (pike, pike-perch, perch, saithe, and cod) versus lean meat (beef and pork)	RCT parallel 8 weeks	33 patients with coronary heart disease	100–150 g fish 4 meals/week	(i) 7.1 and 9.1% ↓ in fasting serum apoA-I concentrations comparing lean fish with lean meat or fatty fish consumption^*∗*^

Isherwood et al. (2010) [84]	Prawns versus crab sticks	RCT crossover 12 weeks	23 healthy males	225 g/day	(i) No differences in fasting plasma apoA-I concentrations

^*∗*^Percentages calculated from the mean values; EPA: eicosapentaenoic acid, DHA: docosahexaenoic acid, SFA: saturated fatty acids, and ALA: alpha lipoic acid.

**Table 5:** Effect of fiber on fasting apoA-I concentrations.

First author, year	Food component/product	Study design and duration	Participants	Intake	Effect
Anderson et al. (1991) [85]	Oat versus wheat bran diet (similar macronutrient composition)	RCT parallel 3 weeks	20 hypercholesterolemic men	14 g/day	(i) No differences in serum apoA-I concentrations

Cara et al. (1992) [86]	Oat versus rice versus wheat bran versus wheat germ (similar macronutrient composition)	RCT crossover 1 day	6 healthy subjects	10 g as oat, rice, and wheat bran versus 4.2 g as wheat germ	(i) No differences in serum apoA-I concentrations

Kashtan et al. (1992) [87]	Oat versus wheat bran supplemented food	RCT parallel 2 weeks	32 subjects with a history of polypectomy and 32 healthy subjects	6.8 g/1000 kcal/day	(i) No differences in serum apoA-I concentrations

Stewart et al. (1992) [80]	Oat bran versus control diet (similar macronutrient composition)	RCT crossover 6 weeks	24 hypercholesterolemic subjects	50 g/day	(i) No differences in serum apoA-I concentrations

Uusitupa et al. (1992) [88]	Oat versus wheat bran diet (similar macronutrient composition)	RCT parallel 8 weeks	36 hypercholesterolemic subjects	10.3 g/day	(i) No differences in serum apoA-I concentrations

Zhang et al. (1992) [89]	Oat versus wheat bran	RCT crossover 3 weeks	9 subjects with ileostomies	4.9 versus 29.0 g/day	(i) No differences in plasma apoA-I concentrations

Mekki et al. (1997) [90]	High-fiber diet versus low fiber diet	RCT parallel 4 weeks	31 mildly hypercholesterolemic subjects	35 g/day	(i) No differences in plasma apoA-I concentrations

Jenkins et al. (2002) [91]	Low fat, low-cholesterol diet high versus low in *β*-glucan or psyllium fiber (similar macronutrient composition)	RCT crossover 4 weeks	68 hyperlipidemic subjects	8 g/day	(i) 1.3% ↓ in serum apoA-I concentrations comparing the high with the low fibers^*∗*^

Garcia et al. (2006) [92]	Arabinoxylan supplement versus placebo	RCT crossover 6 weeks	11 patients with impaired glucose tolerance	15 g/day	(i) 4.0% ↓ in serum apoA-I concentrations^*∗*^

Sola et al. (2007) [93]	Low SFA diet supplemented with P. ovata husk versus *P*. *ovata* seeds	RCT crossover 8 week	28 men with CVD	10.5 g/day	(i) No differences in fasting plasma apoA-I concentrations

Rondanelli et al. (2008) [94]	*β*-Glucan versus rice bran supplemented food	RCT crossover 4 weeks	24 mildly hypercholesterolemic men	15 versus 30 g/day	(i) No differences in serum apoA-I concentrations

Dodin et al. (2008) [95]	Flaxseed versus wheat germ	RCT parallel 12 months	199 healthy postmenopausal women	40 g/day	(i) 4.0% ↓ in serum apoA-I concentrations comparing flaxseed with wheat germ^*∗*^

^*∗*^Percentages calculated from the mean values; SFA: saturated fatty acids.

**Table 6:** Effect of different nuts on fasting apoA-I concentrations.

First author, year	Food component/product	Study design and duration	Participants	Intake	Effects
Munoz et al. (2001) [98]	Walnuts versus Mediteranean cholesterol-lowering diet (similar macronutrient composition)	RCT crossover 6 weeks	10 hypercholesterolemic men	41–56 g/day	(i) No differences in serum apoA-I concentrations

Rajaram et al. (2009) [99]	Walnut versus control diet	RCT crossover 4 weeks	25 mildly hyperlipidemic subjects	42.5 g/day	(i) No differences in serum apoA-I concentrations

Aronis et al. (2012) [97]	Walnut versus control diet (similar macronutrient composition)	RCT crossover 4 days	15 patients with metabolic syndrome	48 g/day	(i) 8.1% ↑ in serum apoA-I concentrations comparing walnut with control diet^*∗*^

Sabaté et al. (2003) [101]	Diet without versus low versus high in almonds	RCT crossover 4 weeks	25 healthy subjects	0, 10, and 20 energy%	(i) No differences in serum apoA-I concentrations

Li et al. (2011) [100]	Almond versus NCEP^1^ diet	RCT crossover 12 weeks	20 patients with diabetes mellitus type 2	60 g/day	(i) No differences in plasma apoA-I concentrations

Mercanligil et al. (2007) [102]	Hazelnut versus low fat, low-cholesterol high-carbohydrate diet	Period 1 control and period 2 intervention 4 weeks	15 hypercholesterolemic men	40 g/day	(i) No differences in plasma apoA-I concentrations

Tey et al. (2011) [103]	Ground versus sliced versus whole hazelnuts	RCT crossover 4 weeks	48 mildly hypercholesterolemic subjects	30 g/day	(i) No differences in plasma apoA-I concentrations

Sheridan et al. (2007) [104]	Pistachio versus control diet (similar macronutrient composition)	RCT crossover 4 weeks	15 mildly hypercholesterolemic subjects	56–85 g/day	(i) No differences in serum apoA-I concentrations

^*∗*^Percentages calculated from the mean values; ^1^NCEP: National Cholesterol Education Program step II.

**Table 7:** Effect of plant sterols on fasting apoA-I concentrations and apoA-I kinetics.

First author, year	Food component/product	Study design and duration	Participants	Intake	Effects
Gylling and Miettinen (1994) [105]	Sitostanol versus control margarine	RCT crossover 6 weeks	11 hypercholesterolemic diabetic men	3.0 g/day	(i) 4.3% in fasting serum apoA-I concentrations^*∗*^ (ii) No difference in apoA-I FCR

Temme et al. (2001) [106]	Plant sterols enriched versus nonplant sterol enriched margarine	RCT crossover 4 weeks	42 mildly hypercholesterolemic subjects	2.0 g/day	(i) No differences in serum apoA-I concentrations

Amundsen et al. (2002) [107]	Plant sterol esters versus control with similar fatty acid composition	RCT crossover 8 weeks	38 children with familial hypercholesterolemia	1.6 g/day	(i) No differences in plasma apoA-I concentrations

Chan et al. (2007) [108]	Olive oil versus sunflower oil with plant sterols versus olive oil with plant sterols margarine	RCT crossover 4 weeks	21 moderately overweight, hypercholesterolemic subject	70% of total fat in the diet 1.7 g plant sterols/day	(i) 0.8% ↑ in fasting plasma apoA-I concentrations comparing olive oil with plant sterols with olive oil alone or sunflower oil with plant sterols^*∗*^

Madsen et al. (2007) [109]	Plant sterols versus control with similar fatty acid composition	RCT crossover 4 weeks	46 mildly hypercholesterolemic subjects	2.3 g/day	(i) No differences in serum apoA-I concentrations

Ooi et al. (2007) [110]	Plant sterols enriched versus nonplant sterol enriched margarine and cereals	RCT crossover 4 weeks	9 patients with the metabolic syndrome	2.0 g/day	(i) No differences in plasma apoA-I concentrations (ii) No effect on apoA-I PR (iii) No effect on apoA-I FCR

Hernández-Mijares et al. (2010) [111]	Healthy diet (NCEP)^1^ versus healthy diet with plant sterols versus normal diet with plant sterols	RCT parallel 3 months	84 mildly hypercholesterolemic subjects	2.0 g/day	(i) 4.0% ↑ in serum apoA-I concentration comparing prudent diet with plant sterols with prudent diet alone or normal diet with plant sterols^*∗*^

Söderholm et al. (2010) [112]	Rye bread with low versus high versus no plant sterols	RCT parallel 2 weeks	68 healthy subjects	2.0 versus 4.0 g/day	(i) No differences in serum apoA-I concentrations

Gagliardi et al. (2010) [113]	Plant sterol margarines versus no-trans-FA margarine versus butter	RCT parallel 5 weeks	53 subjects with metabolic syndrome	2.4 g/day	(i) No differences in plasma apoA-I concentrations

Sialvera et al. (2012) [114]	Yogurt beverage with versus without phytosterol	RCT parallel 2 months	108 patients with the metabolic syndrome	4.0 g/day	(i) No differences in plasma apoA-I concentrations

^*∗*^Percentages calculated from the mean values; ^1^NCEP: National Cholesterol Education Program.

**Table 8:** Effect of soy protein or isoflavone in soy on apoA-I concentrations.

First author, year	Food component/product	Study design and duration	Participants	Intake	Effect
Bakhit et al. (1994) [115]	Soybean protein with or without soybean fiber	RCT crossover 4 weeks	21 hypercholesterolemic men	25 g/day	(i) No differences in fasting plasma apoA-I concentrations

Kurowska et al. (1997) [116]	Soy protein versus milk protein	RCT crossover 4 weeks	34 hypercholesterolemic subjects	2% of daily intake	(i) No differences in fasting plasma apoA-I concentrations

Nilausen and Meinertz (1998) [117]	Soy protein versus casein (similar macronutrient composition)	RCT crossover 1 month	9 healthy men	154 g/day	(i) 10.7% ↑ in fasting plasma apoA-I concentrations^*∗*^

Jenkins et al. (2000) [118]	Soy incorporated into breakfast cereals versus no soy	RCT crossover 3 weeks	25 hyperlipidemic subjects	36 g/day	(i) No differences in fasting serum apoA-I concentrations

Chen et al. (2006) [119]	Soy protein versus milk protein	RCT parallel 3 months	26 patients undergoing hypercholesterolaemic hemodialysis	30 g/day	(i) No differences in fasting serum apoA-I concentrations

McVeigh et al. (2006) [120]	Soy protein varying in isoflavone content	RCT crossover 57 days	35 healthy young men	1.64–61.7 mg isoflavone/day	(i) No differences in fasting serum apoA-I concentrations

Pipe et al. (2009) [121]	Soy protein isolate versus milk protein isolate	RCT crossover 57 days	29 patients with diabetes mellitus type 2	80 g/day	(i) No differences in fasting serum apoA-I concentrations

Campbell et al. (2010) [122]	Soy protein products versus casein products	RCT parallel 1 year	62 moderately hypercholesterolemic postmenopausal women	25 g/day	(i) 8.5% ↓ in fasting serum apoA-I concentrations^*∗*^

Tabibi et al. (2010) [123]	Soy protein versus no soy protein	RCT parallel 8 weeks	40 peritoneal dialysis patients	28 g/day	(i) No differences in fasting serum apoA-I concentrations

Jenkins et al. (2002) [124]	High versus low isoflavone soy protein	RCT crossover 1 months	41 hyperlipidemic subjects	Soy: 50–52 g/day; isoflavones: 73 versus 10 mg/day	(i) No differences in fasting serum apoA-I concentrations

Cicero et al. (2002) [125]	Soy proteins supplemented with isolated *β*-sitosterol versus no soy protein	RCT parallel 40 days	20 moderately hypercholesterolemic subjects	10 g/day	(i) No differences in fasting plasma apoA-I concentrations

Matthan et al. (2007) [126]	Different sources of soy protein versus animal protein	RCT crossover 6 weeks	28 hypercholesterolemic subjects	6.8–7.5 energy%/day	(i) 2.0% ↑ in fasting plasma apoA-I concentrations comparing soy-milk with soybean and soy flour (ii) No differences in fasting plasma apoA-I concentrations comparing soy with animal protein

Bakhtiary et al. (2012) [127]	Soy protein versus soy nuts versus no soy	RCT parallel 3 months	75 women with the metabolic syndrome	35 g/day	(i) 18.8% ↑ in fasting serum apoA-I concentrations comparing soy protein with control^*∗*^ (ii) 25.0% ↑ in fasting serum apoA-I concentrations comparing soy nuts with control^*∗*^

Wangen et al. (2001) [128]	No versus low versus high soy isoflavone	RCT crossover 3 months	18 mildly hypercholesterolemic women	7.1, 65, 132 mg/day	(i) No differences in fasting plasma apoA-I concentrations

Santo et al. (2010) [129]	Milk protein versus isoflavone-poor soy versus isoflavone-rich soy	RCT crossover 28 days	30 healthy young men	25 g protein/day	(i) No differences in fasting and postprandial plasma apoA-I concentrations

^*∗*^Percentages calculated from the mean values.

**Table 9:** Effect of other food components or products on apoA-I concentrations.

First author, year	Food component/product	Study design and duration	Participants	Intake	Effects
Sacks et al. (1984) [130]	Eggs versus no foods containing eggs	RCT crossover 3 weeks	17 healthy subjects	400 kcal/day	(i) No differences in fasting plasma apoA-I concentrations

Luley et al. (1986) [131]	Dried garlic versus control	RCT crossover 6 weeks	(i) 34 hyperlipidemic patients	3 × 198 mg/day	(i) Both no differences in fasting serum apoA-I concentrations

Luley et al. (1986) [131]	Dried garlic versus control	RCT crossover 6 weeks	(i) 51 hyperlipidemic patients	3 × 450 mg/day	(i) Both no differences in fasting serum apoA-I concentrations

Hughes et al. (1994) [132]	Beta-carotene versus wheat germ oil capsules	RCT parallel 30 days	59 hyperlipidemic patients 36 healthy subjects	300 mg/day	(i) No differences in fasting serum apoA-I concentrations

Nanjee et al. (1996) [133]	Glucosinolate free vegetable versus Brussels sprouts	RCT parallel 3 weeks	10 healthy men	300 g/day	(i) No differences in fasting plasma apoA-I concentrations

Nanjee et al. (1996) [133]	Eugenol versus placebo capsule	RCT parallel 3 weeks	10 healthy men	150 mg/day	(i) No differences in fasting plasma apoA-I concentrations

Neil et al. (1996) [134]	Dried garlic versus placebo powder	RCT parallel 6 months	115 hypercholesterolemic subjects	900 mg/day	(i) No differences in fasting serum apoA-I concentrations

Itoh et al. (1997) [135]	Magnesium versus placebo supplement	RCT parallel 4 weeks	33 healthy subjects	411–548 mg/day	(i) No differences in fasting serum apoA-I concentrations

Guimarães et al. (2000) [136]	Eggplant versus placebo powder	RCT parallel 5 weeks	38 hypercholesterolemic subjects	12 g powder/day, corresponded with 100 g eggplant/day	(i) No differences in fasting serum apoA-I concentrations

Oosthuizen et al. (2000) [137]	Dry beans versus no beans	RCT crossover 4 weeks	22 hyperlipidemic patients	110 g/day	(i) No differences in fasting serum apoA-I concentrations

Gammon et al. (2012) [138]	Healthy diet with versus without green kiwifruits	RCT crossover 4 weeks	85 hypercholesterolemic men	2 kiwifruits/day	(i) No differences in fasting serum apoA-I concentrations

Mullan et al. (2016) [139]	Polyphenols versus control	RCT parallel 4 weeks	20 healthy overweight or obese subjects	250 ml (361 mg) polyphenols + 120 mg vitamin C	(i) No differences in fasting plasma apoA-I concentrations

Ohlsson et al. (2010) [140]	Sphingolipids versus placebo	RCT parallel 1 day	18 healthy men	40 g high fat meal 975 mg milk sphingolipids	(i) No differences in postprandial plasma apoA-I concentrations

Castilla et al. (2006) [141]	Red grape juice versus no juice	RCT parallel 2 weeks	26 hemodialysis patients 12 hemodialysis control patients 15 healthy subjects	100 ml/day	(i) 13.2% ↑ in fasting plasma apoA-I concentrations comparing juice with no juice consumption in hemodialysis patients^*∗*^ (ii) 63.2% ↑ in fasting plasma apoA-I concentrations comparing juice consumption in healthy subjects with no juice consumption in hemodialysis patients^*∗*^

Roza et al. (2007) [142]	Citrus flavonoids with tocotrienols versus placebo	RCT parallel 12 weeks	120 hypercholesterolemic subjects	270 mg citrus flavonoids + 30 mg tocotrienols/day	(i) 5.0% ↑ in fasting plasma apoA-I concentrations

Salehpour et al. (2012) [143]	Vitamin D3 versus control supplement	RCT parallel 12 weeks	77 healthy overweight or obese subjects	25 mg/day	(i) 9.2% ↑ in fasting serum apoA-I concentrations^*∗*^

Heravifard et al. (2013) [144]	Vitamin D versus calcium and vitamin D versus control	RCT parallel 12 weeks	90 patients with diabetes mellitus type 2	150 mg calcium versus150 mg calcium and 500 IU vitamin D versus 250 mg calcium and 500 IU vitamin D	(i) 18% ↑ in fasting serum apoA-I concentrations comparing vitamin D with control^*∗*^ (ii) 16% ↑ in fasting serum apoA-I concentrations comparing vitamin D with calcium with control^*∗*^

Neufingerl et al. (2013) [145]	Theobromine versus placebo	RCT parallel 4 weeks	152 healthy subjects	0, 150, 850, and 1000 mg/day	(i) 7.6% ↑ in fasting serum apoA-I concentrations comparing 850 mg theobromine with placebo^*∗*^

Constans et al. (2015) [146]	Orange juice versus control	RCT crossover 4 weeks	25 male subjects with 2 CVD risk factors	3 × daily 200 ml	(i) 6.2% ↑ in fasting plasma apoA-I concentrations comparing orange juice with placebo^*∗*^

Han et al. (2016) [147]	Low versus high dose of grape pomace and omija fruit versus control	RCT parallel 10 weeks	76 healthy overweight or obese subjects	342.5 versus 685.0 mg grape pomace/day and 57.5 versus 115.0 mg omija/day	(i) 10% ↑ in fasting plasma apoA-I concentrations after the high dose compared with control^*∗*^ (ii) No difference in fasting plasma apoA-I concentrations after the low dose compared with control

^*∗*^Percentages calculated from the mean values.

**Table 10:** Summary of the pharmacological approaches targeting apoA-I metabolism in humans.

First author, year	Infusion	Duration	Model	Dose	Effect
Bloedon et al. (2008) [148]	D-4F	A single dose	Coronary artery diseased patients	30 versus 100 versus 300 versus 500 mg	(i) ↑ anti-inflammatory activity of HDL

Nissen et al. (2003) [149]	ApoA-I Milano	One infusion for 5 weeks	Patients with acute coronary syndromes	15 versus 45 mg/kg	(i) 15.1 mm^3^ and 12.6 mm^3^↓ in atheroma volume

Kempen et al. (2016) and Kallend et al. (2016) [150, 151]	ApoA-I Milano	5 doses during 2 hours	Patients with stable coronary artery disease	10 versus 20 versus 30 versus 40 mg/kg	(i) Dose-dependent ↑ in apoA-I concentrations (ii) Dose-dependent shift from small- to large-sized HDL particles. (iii) ↑ in ABCA1-mediated cholesterol efflux

Nicholls (2016) [152]	ApoA-I Milano	5 weekly doses	120 patients with a recent acute coronary syndrome	20 mg/kg	(i) 7.8 and 5.3% ↓ in fasting HDL-C and apoA-I concentrations (ii) No effects on percent and total atheroma volume

Nicholls (2007) [152]	CSL-111	Once a week for a month	Patients elected for coronary angiography	40 versus 80 mg/kg	(i) Abnormalities in liver function test (ii) 3.4% ↓ in atheroma volume

Easton et al. (2014) and Gille et al. (2014) [153, 154]	CSL112	A single dose	Healthy volunteers	5 versus 15 versus 40 versus 70 versus 105 versus 135 mg/kg	(i) ↑ in apoA-I concentrations for 3 days or longer (ii) 81% ↑ in HDL concentrations (iii) ↑ pre-*β*-HDL particle concentrations (iv) 2.9-fold ↑ in cholesterol efflux

Easton et al. (2014) and Gille et al. (2014) [153, 154]	CSL112	Once or twice weekly for 4 weeks	Healthy volunteers	3.4 versus 6.7 g once a week versus 3.4 g twice a week	(i) ↑ in apoA-I concentrations for 3 days or longer (ii) ↑ pre-*β*-HDL particle concentrations (iii) 2.6-fold ↑ in cholesterol efflux

Tricoci et al. (2015) [155]	CSL112	A single dose	Patients with atherosclerosis	1.7 versus 3.4 versus 6.8 g	(i) No elevations in alanine aminotransferase or aspartate aminotransferase (ii) No serious adverse events. (iii) Dose-dependent ↑ in apoA-I concentrations and total cholesterol efflux

Gibson et al. (2016) [156]	CSL112	4 weekly infusions	Patients with myocardial infarction	0 versus 2 versus 6 g	(i) Safe for use (ii) Dose-dependent ↑ in fasting apoA-I, HDL-C concentrations and cholesterol efflux

Tardif (2014) [157]	CER-001	6 weekly infusions	Patients with acute coronary syndromes	3 versus 6 versus 12 mg/kg	(i) No changes in atheroma volumes

Kootte et al. (2015) [158]	CER-001	9 infusions twice weekly for 28 days	Patients with familial hypoalphalipoproteinemia	8 mg/kg	(i) 94% ↑ in apoA-I concentrations (ii) 117% ↑ in HDL-C concentrations (iii) 8.8% ↓ atherosclerotic lesion size (iv) 44% ↑ in cholesterol efflux (v) ↑ fecal neutral sterol excretion

Hovingh et al. (2015) [159]	CER-001	12 infusions twice weekly	Patients with homozygous familial hypercholesterolemia	8 mg/kg	(i) 13% ↑ in apoA-I concentrations^*∗*^ (ii) 2.8% ↓ in vessel wall area^*∗*^ (iii) Trend toward ↓ in vessel wall thickness

Zheng et al. (2016) [160]	CER-001	A single dose	Patients with atherosclerotic carotid artery disease	3 mg/kg	(i) 8.7% ↑ in apoA-I concentrations^*∗*^ (ii) 13.8% ↑ in the cholesterol efflux capacity

Nicholls et al. 2017 [161]	CER-001	10 weekly infusions	Coronary artery diseased patients		(i) No difference in atheroma volume (ii) No difference in LDL-C

Bloedon et al. (2008) [148]	D-4F	A single dose	Coronary artery diseased patients	30 versus 100 versus 300 versus 500 mg	(i) ↑ anti-inflammatory activity of HDL

Bailey et al. (2010) [162]	RVX-208	7 days	Healthy subjects	1 to 20 mg/kg/day	(i) 11% ↑ in apoA-I concentrations (ii) 11% ↑ in HDL-C concentrations (iii) 42% ↑ in pre-*β*1-HDL concentrations (iv) 11% ↑ in ABCA1-mediated cholesterol efflux

Nicholls et al. (2011) [163]	RVX-208	Twice daily for 12 weeks	Patients with stable coronary artery disease	50 versus 100 versus 150 mg	(i) No difference in apoA-I concentrations

Gilham et al. (2016) [164]	RVX-208	24 weeks	Statin-treated patients with low HDL-C concentrations	200 mg/day	(i) ↑ in apoA-I concentrations (ii) ↑ HDL particle number (iii) Safe for oral use

Nicholls et al. (2016) [32]	RVX-208	26 weeks	Statin-treated patients with coronary artery disease and low HDL-C concentrations	100 mg twice daily	(i) No difference in atheroma volume, HDL-C, and apoA-I concentrations

Siebel et al. (2016) [165]	RVX-208	29–33 days	20 males with prediabetes	100 mg	(i) No change in HDL-C and apoA-I concentrations (ii) 11% ↑ in medium size HDL particles (iii) 10% ↓ in small size HDL particles (iv) Later and ↑ glucose peak (v) ↓ endogenous glucose production

Shamburek et al. (2016) [166]	Recombinant human lecithin-cholesterol acyltransferase infusion	7 months	1 patient with familial lecithin-cholesterol acyltransferase deficiency	Optimization phase: 3 times, 1 hour, 0.3, 3.0, and 9.0 mg/kg. Maintenance phase: every 1 to 2 weeks, 3.0 or 9.0 mg/kg	(i) ↑ apoA-I, HDL-C, and to a lesser extent LDL-C (ii) ↑ postprandial triacylglycerol concentrations

^*∗*^Percentages calculated from the mean values.
